# Microscopy in Mitochondrial Research: A Comprehensive Review

**DOI:** 10.1242/jcs.263933

**Published:** 2026-04-01

**Authors:** Prasanna Katti, Prasanna Venkhatesh, C Nivedya, Andrea Marshall, Amber Crabtree, Zahra Elly Soltani, Olga Korolkova, Vineeta Sharma, Suraj Thapliyal, Karthik Naik, Kit Neikirk, Rodolpho Ornitz Oliveira Souza, Sepiso K. Masenga, Nelson Wandira, Magdalene Ameka, Okwute M. Ochayi, Christian A. Combs, Lucy Collinson, Nalan Liv, Antentor Hinton

**Affiliations:** 1 Department of Biology, Indian Institute of Science Education and Research Tirupati (IISERT), Tirupati, AP, India, 517619; 2 Department of Molecular Physiology and Biophysics, Vanderbilt University, Nashville, TN, 37232, USA; 3 Department of Biochemistry, Cancer Biology, Neuroscience and Pharmacology, Meharry Medical College, Nashville, TN 37208, USA; 4 Department of Physiological Sciences, School of Medicine and Health Sciences, Mulungushi University, Livingstone, Zambia, 10101; 5 Institute of Health Sciences, Busoga University, Iganga, Uganda; 6 Department of Medical Microbiology and Immunology, KAVI-ICR, University of Nairobi, Nairobi, Kenya; 7 Department of Physiology, Faculty of Basic Medical Sciences, Baze University, FCT-Abuja, Nigeria, 900001; 8 Light Microscopy Core, NHLBI, National Institutes of Health, Maryland, Bethesda, USA; 9 Electron Microscopy Science Technology Platform, Francis Crick Institute, 1 Midland Road, London NW1 1AT, UK; 10 Department of Cell Biology, Center for Molecular Medicine, University Medical Center Utrecht, Heidelberglaan 100, 3584 CG Utrecht, the Netherlands; 11 Department of Microbiology and Immunology, Jacobs School of Medicine and Biomedical Sciences, University at Buffalo (SUNY), Buffalo, NY, USA; 12 The First Center for Autism and Innovation, Vanderbilt University, Nashville, Tennessee, USA; 13 Department of Physics and Astronomy, Vanderbilt University, Nashville, Tennessee, USA

**Keywords:** Mitochondria, Confocal, Super-Resolution Microscopy, EM, CLEM, Volume EM

## Abstract

Mitochondria are highly dynamic, double-membrane organelles that play integral roles beyond energy production. Mitochondria adapt their morphology to meet diverse cellular demands, and highly plastic mitochondrial networks interact and communicate with various cellular components to maintain cellular health. Advances in both light and electron microscopy have greatly enhanced our understanding of mitochondrial structure and function. However, the small diameter of mitochondrial tubules, often near the diffraction limit of light, poses challenges for visualizing submitochondrial structures and protein distributions with conventional microscopy. Recently, super-resolution microscopy has offered unprecedented insights into mitochondrial dynamics, interactions, and architecture. In this Review, we discuss how imaging techniques have advanced our understanding of mitochondrial biology. We critically assess the contributions of 2D electron microscopy to elucidating the native architecture of cristae and respiratory chain complexes. Additionally, we explore how 3D electron microscopy and super-resolution methods have reshaped our comprehension of mitochondrial network dynamics, heterogeneity, and interactions with other cellular components. Finally, we discuss the strengths and limitations of various approaches, considering their potential to overcome current challenges and open new avenues in mitochondrial research, and illuminate how advanced microscopy continues to drive discoveries in mitochondrial biology with implications for metabolic diseases and aging.

## Introduction

Since mitochondria were first characterized by Richard Altmann in 1890, and later named by Carl Benda in 1898 (Ernster and Schatz, 1981), they have remained a focal point in biological research due to their central role in metabolism. However, mitochondria are submicron organelles with highly organized internal structure (typically 0.5–1 µm in diameter and 1-10 µm in length, with inner-membrane cristae and cristae junctions on the order of 10-40 nm). C onventional light microscopy, limited by the physics of light diffraction, cannot resolve structures smaller than ∼200 nm, rendering it insufficient for detailed studies of mitochondrial structure and function (Schermelleh et al., 2010). Early electron microscopy (EM) studies in the 1950s and 1960s provided the first detailed views of mitochondrial ultrastructure, revealing the distinctive outer and inner mitochondrial membranes (OMM and IMM, respectively) and the intricate folds of the IMMs known as cristae (Palade, 1952; Sjostrand, 1953). Although traditional EM offers high resolution, it provides only static, two-dimensional images of fixed samples, unable to capture mitochondrial dynamics in living cells (Frey and Mannella, 2000). Live-cell imaging techniques developed in the 1980s and 1990s revolutionized mitochondrial research by revealing their highly dynamic (Bereiter-Hahn and Voth, 1994). The development of fluorescent probes, genetic labeling techniques, and confocal microscopy allowed researchers to observe mitochondrial movement, fusion, and fission in living cells (Chen et al., 2003), revealing that mitochondria form complex, interconnected networks that continuously remodel in response to cellular demands and stress (Westermann, 2010). The ability to visualize these dynamics has been instrumental in elucidating the molecular mechanisms governing mitochondrial behavior and their implications for cellular health and disease (Chan, 2012).

In recent years, super-resolution light microscopy methods, such as stimulated emission depletion (STED) microscopy, photoactivated localization microscopy (PALM) and stochastic optical reconstruction microscopy (STORM), have overcome the diffraction limit, enabling visualization of mitochondrial substructures and protein distributions in living cells (Jakobs and Wurm, 2014; Jans et al., 2013; Palmer et al., 2021; Rust et al., 2006). Concurrently, advances in EM, particularly three-dimensional EM (3D-EM) techniques like electron tomography (ET) and focused ion beam-scanning EM (FIB-SEM), have enabled high-resolution 3D reconstructions of mitochondrial ultrastructure (Perkins et al., 1997). These methods have been crucial in elucidating the complex architecture of cristae and their dynamic remodeling in response to cellular signals (Mannella, 2006). Integration of different microscopy techniques has proven particularly powerful; for example, correlative light and electron microscopy (CLEM) combines the labeling specificity and live-cell imaging capabilities of fluorescence microscopy with the ultrastructural detail provided by EM, offering a more comprehensive view of mitochondrial structure and function (de Boer et al., 2015).

This Review explores how these recent advances in microscopy techniques have transformed our understanding of mitochondrial biology. We focus on their impact on the study of mitochondrial network dynamics, cristae ultrastructure, mitochondria–ER contact sites, and the structural basis of mitochondrial function. Furthermore, we evaluate the strengths and limitations of different microscopy modalities used in mitochondrial research (Box 1). We conclude by examining future directions and highlighting how emerging imaging innovations may redefine the frontiers of mitochondrial investigation.

### Fluorescence microscopy techniques in mitochondrial research

#### Confocal and multiphoton microscopy

**Fig. 1:**
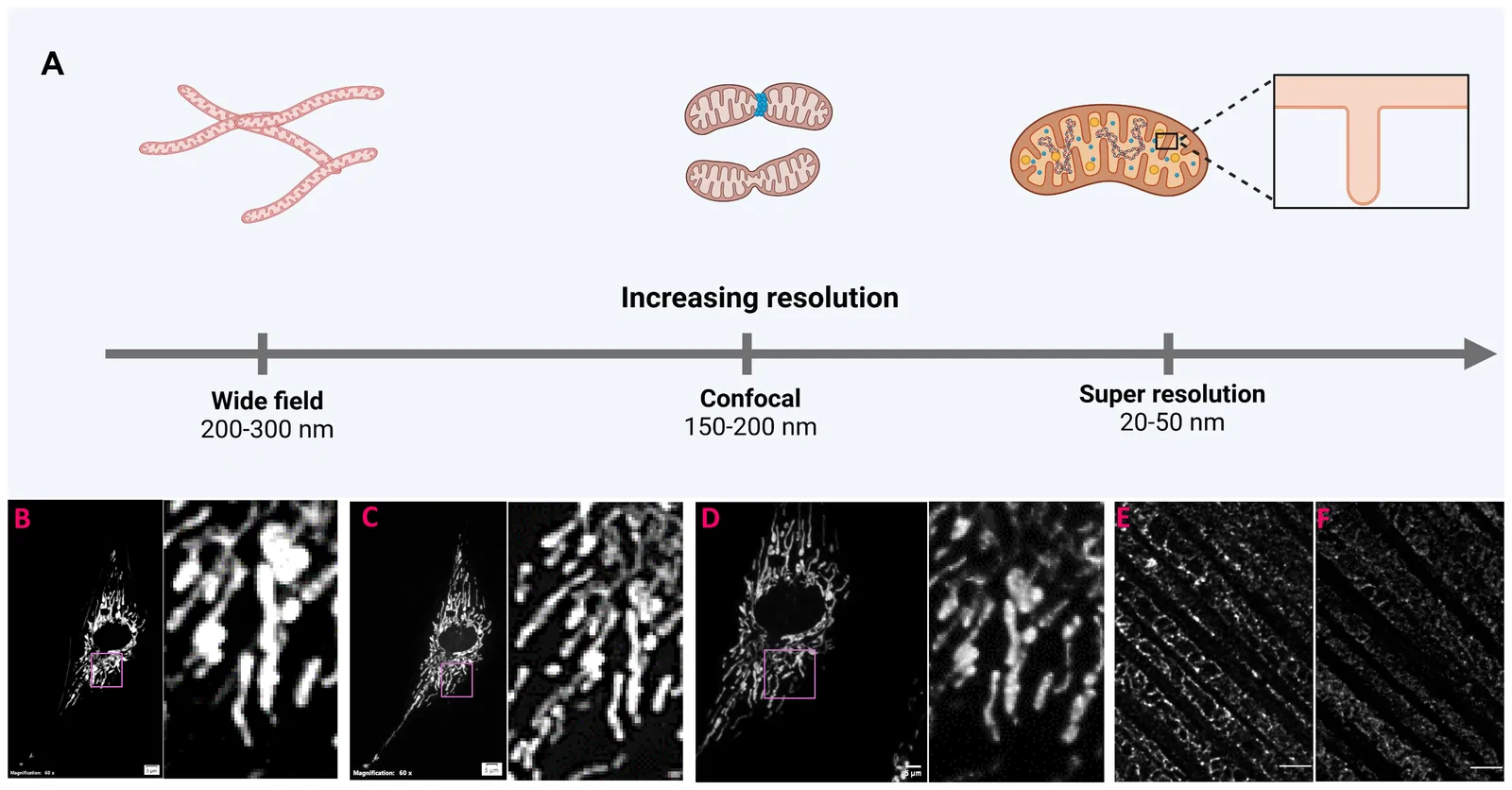
Multi-scale microscopy of mitochondria. (A) Schematic overview showing the range of resolution and visible mitochondrial features across different imaging modalities. (B-D) Mitochondrial imaging in live HeLa cells using MitoTracker™ Red FM across three modalities: (B) wide-field fluorescence, (C) confocal, and (D) super-resolution microscopy. Scale bar: 5 μm. (E-F) Super-resolution STED microscopy of cardiomyocytes showing defined localization of two inner mitochondrial membrane proteins. (E) Cells were immunostained with primary antibodies against ATPβ (ThermoFisher A-21351, 1:200) and (F) CHCHD3 (Millipore Sigma HPA042935, 1:200). Images were acquired using a Leica SP8 STED microscope with XY and Z depletion, then deconvolved using Huygens software. Scale bar: 2 μm. Created in BioRender. Katti, P. (2026) https://BioRender.com/hitcuun

Confocal laser scanning microscopy (CLSM), which employs a focused laser beam together with a pinhole to eliminate out-of-focus light, resulting in sharper images with better contrast, offers several advantages over conventional widefield fluorescence microscopy, including improved resolution, optical sectioning capability and three-dimensional imaging of living cells (Fig. 1 A-C) (Conchello and Lichtman, 2005; Elliott, 2020 ; Schatten and Pawley, 1988). Decades of key studies of mitochondrial function have used CLSM, for example, to reveal the dynamic nature of mitochondrial Ca^2+^ signaling, demonstrating rapid and localized Ca^2+^ uptake by mitochondria in response to cellular stimuli (Rizzuto et al., 1998), and to visualize mitochondrial membrane potential fluctuations in cardiomyocytes, revealing spontaneous depolarization events that were previously unobservable (Eisner et al., 2017). Spinning disk confocal systems, which employ a rotating disk containing thousands of precisely arranged pinholes and microlenses to enable parallel illumination and detection, have further improved the temporal resolution of confocal microscopy for mitochondrial imaging. Unlike conventional point-scanning confocal systems that sequentially sample individual locations, the parallel architecture of spinning disk systems achieves 10-100× faster acquisition rates while maintaining optical sectioning, making it valuable for capturing rapid mitochondrial dynamics or assessing mitochondrial quantity, morphology, and membrane potential (Ahmadian et al., 2024).

Multiphoton microscopy is a powerful tool for studying mitochondria in intact tissues and organisms. Multiphoton microscopy uses near-infrared (IR) pulsed lasers to excite fluorophores by simultaneously absorbing multiple lower-energy photons. This approach reduces phototoxicity by confining excitation exclusively to the focal plane, employing longer wavelengths that minimize endogenous absorption, and providing recovery intervals between high-intensity pulses that enable prolonged imaging of mitochondria in intact tissues (Zipfel et al., 2003) Although more expensive than single-photon systems, by using long-wavelength IR excitation light, multiphoton systems enable deeper tissue penetration and reduced autofluorescence, light scattering, photodamage and phototoxicity compared to conventional confocal microscopy (Helmchen and Denk, 2005). Multiphoton microscopy is also superior to single-photon microscopy for imaging endogenous fluorescence of mitochondrial metabolites like NAD(P)H and FAD, because it shifts the stimulation of NAD(P)H autofluorescence from ultraviolet (UV) to IR wavelengths (∼720-800 nm), which also minimizes phototoxicity-associated DNA damage in the nucleus and mitochondria (Youn et al., 2007). A key example of the utility of multiphoton microscopy for imaging mitochondria in intact tissues utilized intravital two-photon microscopy to visualize mitochondrial transport and membrane potential in individual axons of living mice (Breckwoldt et al., 2014). These findings challenged the prevailing view that mitochondrial depolarization invariably triggers mitophagy (mitochondrial degradation via autophagy) by revealing spontaneous mitochondrial depolarization and repolarization events, demonstrating how advanced imaging techniques can uncover previously unobserved phenomena and reshape our understanding of mitochondrial biology.

However, both confocal and multiphoton microscopy are limited by light diffraction, restricting their lateral (x-y) resolution to ∼200-300 nm and axial (z) resolution to ∼500-700 nm (Box 1). This limitation has driven the development of super-resolution microscopy techniques, further expanding our ability to visualize mitochondrial structures and processes.

#### Super-Resolution Microscopy

Super-resolution microscopy techniques have bridged the gap between conventional light microscopy and EM, offering nanoscale resolution while retaining the advantages of being compatible with fluorescence labeling (Fig. 1D-F) and live-cell imaging capabilities (Thapilyal et al., 2026).

#### Stimulated emission depletion (STED) microscopy

STED microscopy builds upon confocal microscopy hardware by incorporating an additional depletion light source alongside the excitation light source. The first laser excites fluorophores within the focal region, while the depletion laser selectively suppresses fluorescence in the periphery via stimulated emission (Klar et al., 2000). The depletion laser forms a doughnut-shaped pattern around the excitation center, suppressing spontaneous emission and collecting only the central fluorescence signal. This technique provides direct, high-resolution imaging without requiring algorithmic processing (Fig. 1 E-F). Initially, STED was limited to use in fixed samples due to a lack of fluorescent probes suitable for live imaging. Liu et al., developed a photostable, low-phototoxicity fluorescent stain for the mitochondrial inner membrane that enables extended multicolor live-cell STED nanoscopy of mitochondrial structure and dynamics with sub-100 nm resolution (Liu et al., 2022).

In fixed cells dual-colour STED microscopy has revealed that mitochondrial nucleoids are significantly smaller than previously thought (∼100 nm in diameter) (Kukat et al., 2011). STED and immunogold EM were also employed to investigate the mitochondrial inner membrane organizing system (MINOS) in fixed cells (Neikirk et al., 2023; Jans et al., 2013). (an example of which can be found in Fig. 1E). A recent study that combined STED nanoscopy with dual-color imaging and HB Mito crimson, an exceptionally photostable, deep-red fluorescent probe well-suited for STED that specifically labels the IMM (Ge et al., 2025), demonstrated that extensive functional and structural interactions exist between mitochondrial DNA (mtDNA) and mitochondria. Dual-color imaging of the IMM and mtDNA, which enabled precise mapping of nucleoid position relative to mitochondrial architecture, revealed that mtDNA preferentially localizes to mitochondrial tips or branch points while maintaining an overall regular spatial distribution within the mitochondrial network (Ren et al., 2024). This study changed our understanding of mtDNA organization, in which nucleoids had previously been suggested to be relatively stationary and uniformly distributed, by showing that mtDNA does not distribute randomly within the mitochondrial network but has a positional preference for dynamically active regions like tips and branch points, and additionally demonstrated that membrane curvature and network remodeling contribute to nucleoid localization. Recently, live-cell STED imaging was also used to investigate the nanoscale organization and dynamics of the mitochondrial calcium uniporter (MCU) complex (Hirtl et al., 2025). Interestingly, this study observed that MCU forms distinct clusters along the IMM, with cluster size and distribution changing in response to cellular Ca^2+^ levels.

Improved fluorescent probes and image processing techniques have also expanded the application of STED in mitochondrial research. MitoTracker^TM^ dyes are widely used for mitochondrial labeling but have limitations, particularly for long-term or high-intensity imaging (Buckman et al., 2001). To address some of these limitations, gentler fluorophores, such as photostable and keto-epoxide-based MitoOrange (PKMO), have been developed (Chen et al., 2024). These fluorophores exhibit lower phototoxicity and less photobleaching under repeated or high-power illumination, making them more suitable for extended live-cell STED imaging. For example, using several mitochondrial membrane potential-dependent probes to distinguish the cristae from the inner boundary membrane (IBM), a recent study used both Airyscan imaging (see Box 1) and STED microscopy to image the IMM with high spatial and temporal resolution in living cells (Wolf et al., 2019). Further improvements in STED technology include precise localization techniques like minimal stimulated emission depletion (MINSTED, discussed further below) (Weber et al., 2021) and deep learning-based image restoration (Ebrahimi et al., 2023). Live-cell STED nanoscopy has revealed the nanoscale architecture and dynamic organization of mitochondrial cristae ridges in living cells (Stephan et al., 2019).

Combined, these advances in STED microscopy have helped to significantly enhance our understanding of mitochondrial–ER contact sites (MERCs). Because STED’s high-resolution multicolor imaging enables the concurrent visualization of multiple mitochondrial components and their interactions with surrounding organelles, it can offer a more integrated perspective on mitochondrial function within the cellular environment (Bottanelli et al., 2016). For example, multi-color STED was combined with parallelized reversible saturable or switchable optical fluorescence transitions (RESOLFT) nanoscopy – a method that uses photo-switchable fluorescent proteins – to study MERC organization and dynamics in neuronal cells (Damenti et al., 2021). Using this approach, which enabled prolonged time-lapse imaging of multiple contact sites, Damenti and colleagues visualized mitochondrial constriction by fine transverse ER tubules throughout the dendrites and axons of the same neuron (Damenti et al., 2021). -Live-cell STED nanoscopy has also been used to visualize ER architecture, revealing an interconnected and highly dynamic network of membrane sheets and tubules, and demonstrating that structures appearing as ER sheets by conventional microscopy often correspond to densely packed regions of rapidly moving ER tubules (Schroeder et al., 2019). Together, these studies illustrate how live-cell super-resolution imaging can resolve dynamic membrane architectures in intact cells, providing a conceptual framework for investigating the nanoscale organization and transient behavior of mitochondria–ER contact sites.

Despite its advantages, STED microscopy still presents notable challenges for mitochondrial imaging, as the high-intensity laser exposure can cause photodamage and artificially alter mitochondrial morphology and function (Wäldchen et al., 2015). These challenges necessitate careful optimization of imaging parameters and the use of specialized fluorophores to mitigate damage while maintaining resolution. In this context, advanced approaches such as Tau-STED (Alvarez et al., 2021) and fluorescence lifetime-encoding strategies have been shown to substantially reduce the required STED depletion power while preserving STED-level resolution. Tau-STED uses fluorescence lifetime information to temporally distinguish between undepleted and depleted emission, enabling effective resolution enhancement at a fraction of the depletion power required in conventional STED (Alvarez et al., 2021). Similarly, lifetime-based encoding and detection schemes such as gated STED, SPLIT, and phasor-based lifetime filtering improve signal separation and contrast, thereby minimizing photobleaching and phototoxicity during high-resolution imaging (Alvarez et al., 2021; Vicidomini et al., 2011; Moffitt et al., 2011; Lanzanò et al., 2015; Wang et al., 2018)

#### Single-Molecule Localization Microscopy (SMLM)

SMLM enables the tracking of fluorescent molecules and their trajectories over time by sequentially imaging multiple on/off cycles (“blinking”) of sparsely activated, non-overlapping fluorophores (Lelek et al., 2021). Stochastic blinking isolates single emitters in time, enabling precise localization of each emitter. SMLM techniques, including photoactivated localization microscopy (PALM) and stochastic optical reconstruction microscopy (STORM), achieve super-resolution by precisely localizing individual fluorescent molecules over numerous activation-deactivation cycles (Betzig et al., 2006; Rust et al., 2006). These ingenious techniques have provided unprecedented insights into the nanoscale organization of mitochondrial proteins.

Several SMLM strategies, along with specialized fluorescent probes and antibodies, have enabled detailed study of mitochondrial components, including the IMM, outer OMM, and mitochondria-associated membranes (MAM) (Jolivet and Bertolin, 2025; Landoni et al., 2024). For example, STORM was used to reveal distinct cluster arrangements of IMM proteins and to detect changes in the organization of complex IV subunits in cells lacking specific mitochondrial chaperones (Palmer et al., 2021). Similarly, direct STORM (dSTORM) imaging was used to visualize the spatial organization of F_0_F_1_-ATP synthase and uncoupling protein 4 (UCP4), which regulates mitochondrial membrane potential (Klotzsch et al., 2015). This study found that UCP4 and F_0_F_1_-ATP synthase are spatially separated within the IMM, thereby eliminating proton competition between them.

Although PALM and STORM offer exceptional spatial resolution, live-cell mitochondrial imaging using these techniques remains challenging due to long acquisition times and high-intensity illumination, which can perturb mitochondrial function (Jakobs et al., 2020). Thus, the utility of PALM and STORM for visualizing mitochondrial dynamics was initially limited. H owever, 0.8-1 second temporal resolution has now been achieved using compressed sensing STORM with noise-correction methods, which allows for a fluorophore density that is ten times higher than that allowed in conventional methods. In compressed sensing, a mathematical approach that allows reconstruction of a super-resolution image from a few data points using smart algorithms to fill in the missing information, a sparse signal can be recovered from highly noisy or incomplete data (Zhu et al., 2012). This approach allows for the capture of rapid events, such as mitochondrial tubulation (Chen et al, 2020). In addition, the use of novel probes, such as vicinal dithiol protein (VDP)-targeting fluorophores, which exhibit spontaneous, reversible blinking under red excitation without requiring specialized imaging buffers or thiol additives, enables “buffer-free” STORM imaging (Zhang et al., 2023; Samanta, 2019). Although these probes and buffer-free STORM workflows have not yet been broadly applied to mitochondrial imaging, their properties make them well-suited for future studies. Buffer-free STORM reduces toxicity and simplifies imaging conditions, which would be advantageous for examining dynamic mitochondrial behaviors in live cells. Some fluorescent arsenical dyes show high specificity toward VDPs, which contain two adjacent cysteine residues that form a specific chemical handle for selective labeling, through covalent arsenic–thiol linkages, enabling selective *in situ* labeling and high-resolution STORM imaging (Griffin et al., 1998; Hoffmann et al., 2010; Shen et al., 2013). These developments offer advantages over STED microscopy, including easier multicolor imaging, reduced phototoxicity, and quantitative analysis of protein numbers and clustering (Chen et al., 2020; Nicovich et al., 2017).

#### Structured illumination microscopy (SIM)

SIM enhances resolution through patterned illumination and computational reconstruction, offering a two-fold improvement over conventional microscopy. Structured illumination refines resolution by transforming unresolvable fine details into visible, low-resolution patterns called moiré fringes, making it easier to detect tiny, hard-to-see structures. In SIM, resolution is improved by illuminating the sample with fine patterns of light that generate detectable interference patterns, allowing spatial information beyond the diffraction limit to be computationally recovered (Gustafsson, 2005). In 3D SIM, the incorporation of a transmission phase grating (an optical element with fine, evenly spaced lines that splits incoming light into several beams) into the illumination pathway diffracts a collimated beam (in which light rays travel in parallel, maintaining a narrow focused path) into three distinct illumination beams directed at one sample, where their interference generates a 3D structured illumination pattern essential for achieving enhanced spatial resolution (Shao et al., 2011).

SIM offers a balance between resolution enhancement and live-cell compatibility and supports multicolor imaging, making it a valuable tool for studying dynamic mitochondrial processes (Chen et al., 2023). It provides more modest resolution than other super-resolution methods, achieving ∼100 nm lateral resolution compared to the 20-50 nm resolution possible with STED, STORM, or PALM. However, unlike STED, which requires high-intensity laser illumination, and STORM and PALM, which rely on photo-switchable fluorophores and long acquisition times, SIM is compatible with conventional fluorophores and offers faster imaging. Opstad et al., 2018; Markwirth et al., 2012; Schermelleh et al., 2017

Two functionally and mechanistically distinct types of mitochondrial fission were recently discovered using SIM (Kleele et al., 2021). Live-cell 3D-SIM was used to investigate the dynamics of mitochondrial DNA nucleoids during cell division, revealing that they undergo coordinated movements and segregation to ensure equal distribution between daughter cells – providing new insights into mitochondrial genome inheritance (Lewis et al., 2016). 3D SIM has also been used to visualize the formation under metabolic stress of mitochondrial-derived vesicles (MDVs) (Opstad et al., 2022; Opstad et al., 2022), which are dynamic small (50-100 nm) vesicles that support mitochondrial quality control, immune signaling and communication by delivering selective mitochondrial cargo to peroxisomes, late endosomes, lysosomes and phagosomes, with roles in aging, heart disease and neurodegeneration (König and McBride, 2024).

Overall, these applications demonstrate that SIM provides a powerful combination of enhanced resolution, low phototoxicity, and rapid imaging, well-suited for capturing fast, transient mitochondrial events. As the need for super-resolution tools continues to grow, SIM’s unique balance of performance and compatibility makes it an indispensable tool for dissecting mitochondrial structure, dynamics, and function in living cells.

#### Minimal Emission Fluxes (MINFLUX)

MINFLUX (Balzarotti et al., 2017) is a single-molecule localization technique that localizes individual fluorescent molecules with nanometer precision by stochastically activating and imaging sparse subsets of fluorophores over time. Like STED, MINFLUX employs a ring-shaped light; however, MINFLUX excites fluorophores within the ring region while leaving the center unaffected, determining the position of emitters by locating where fluorescence emission is minimized and mitigating photobleaching and phototoxicity. Significant advantages of MINFLUX include its ability to operate with brief exposure times, thereby collecting fewer photons and enabling sub-millisecond temporal resolution, as well as achieving sub-5 nm spatial resolution by analyzing the position of minimal emission flux. DNA-PAINT MINFLUX nanoscopy was recently employed to precisely localize three mitochondrial target proteins across three color channels, achieving 3D localization precision of approximately 5.4 nm laterally and 3.1 nm axially (Ostersehlt et al., 2022)

#### Minimal Stimulated Emission Depletion (MINSTED)

MINSTED, like MINFLUX, iteratively localizes molecules by collecting photon information from each imaged position (Weber et al., 2021b). This technique leverages STED’s laser illumination, shifting the ring-shaped suppression light and central excitation light in tandem. As the ring’s center approaches a fluorescent molecule, STED suppression weakens, increasing fluorescence emission and pinpointing the molecule’s location at the position with the highest photon count. MINSTED demonstrates exceptional precision, achieving resolutions as low as 4.7 Å while measuring individual molecule positions. Notably, 2D MINSTED data revealed the nanoscale distribution of MIC60, a core component of the mitochondrial contact site and cristae-organizing system (MICOS) complex (Weber et al., 2021b). An integral membrane protein located in the inner mitochondrial membrane, MIC60 plays a crucial role in organizing cristae architecture and maintaining the structure of crista junctions (Van Laar et al., 2019), narrow tubular openings (typically 28-30 nm in diameter) that connect cristae to the IBM.

### EM in Mitochondrial Research

#### Transmission Electron Microscopy (TEM)

**Fig. 2:**
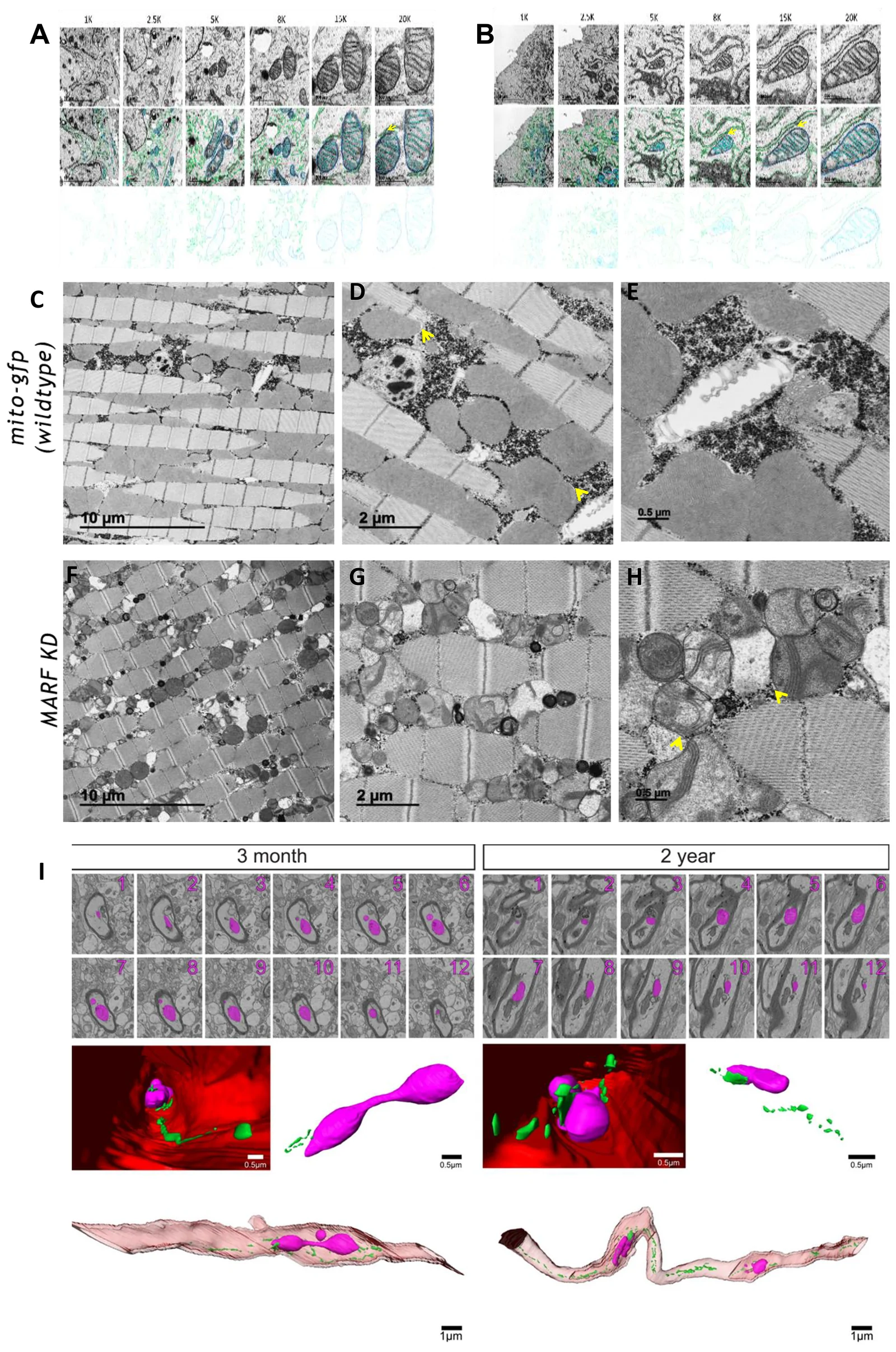
TEM and vEM reveal mitochondrial ultrastructure and organelle interactions (A) Representative 2D transmission electron microscopy (TEM) images of mitochondria–endoplasmic reticulum contact sites (MERCS) in primary myoblasts shown across increasing magnifications (1K, 2.5K, 5K, 8K, 15K, and 20K). The top row displays raw, unaltered TEM images. The bottom row shows the same images with superimposed segmentations highlighting mitochondria (dark blue), cristae (light blue), and endoplasmic reticulum (green), illustrating the progressive resolution of cristae architecture and MERCS with increasing magnification. (B) Representative 2D TEM images of MERCS in primary myoblasts following MFN2 deletion, displayed at identical magnifications (1K–20K). Loss of MFN2 is associated with altered mitochondrial morphology, disrupted cristae organization, and changes in mitochondria–ER juxtaposition compared with control cells. (C–E) 2D TEM images of wild-type *Drosophila melanogaster* skeletal muscle (mito-GFP, WT118) illustrating overall muscle ultrastructure, mitochondrial organization, cristae morphology, and MERCS at progressively higher magnifications. (F–H) Corresponding 2D TEM images from Marf (*Drosophila* MFN homolog) knockdown (KD) skeletal muscle. Marf depletion results in pronounced alterations in mitochondrial size and shape, disrupted cristae architecture, abnormal mitochondria–ER contacts, and accumulation of additional organelles, including lysosomes and glycogen deposits (arrowheads), indicative of altered metabolic and quality-control states. Scale bars: 10 μm (C, F); 2 μm (D, G); 0.5 μm (E, H). (I) 3D electron microscopy reconstruction of mitochondrial inner membrane architecture, highlighting cristae organization. Individual mitochondria are rendered to visualize cristae morphology and spatial complexity at submicron resolution (scale ∼0.5–1 μm). This panel illustrates cristae form derived from high-resolution volumetric EM datasets. (J) Serial block-face scanning electron microscopy (SBF-SEM; vEM) of mouse hypothalamic tissue. *Top:* Representative serial EM sections from 3-month-old (left) and 2-year-old (right) mice. *Bottom:* Corresponding 3D reconstructions of mitochondria (pink) and endoplasmic reticulum (green) within axons (axon interior, red), revealing age-dependent remodeling of mitochondrial morphology, ER distribution, and mitochondria–ER interactions within neuronal processes.

TEM has served as a foundational tool in mitochondrial ultrastructural analysis since the 1950s (Palade 1952). TEM transmits a focused electron beam through ultrathin sections of fixed and stained biological specimens, producing high-resolution images by differentially scattering electrons within cellular components. This allows detailed visualization of mitochondrial architecture at the nanoscale (Palade 1952). Advancements in sample preparation techniques, including high-pressure freezing and freeze-substitution, have markedly improved preservation of mitochondrial ultrastructure for TEM analysis (McDonald and Auer, 2006). These cryo-fixation methods minimize artifacts typically introduced by chemical fixation and dehydration, enabling more faithful representations of mitochondrial morphology (Fig. 2).

**Fig. 3:**
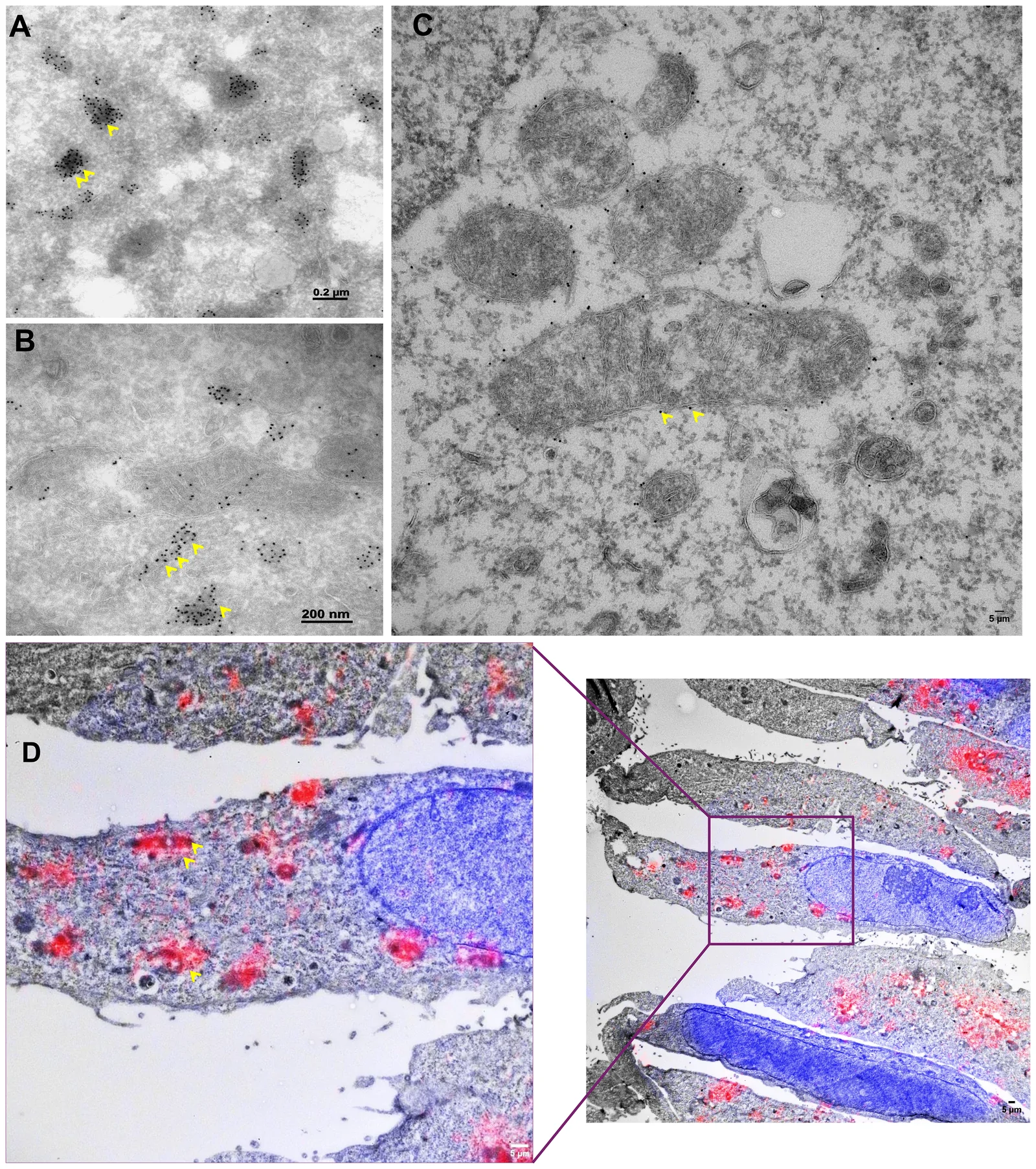
Immunogold EM and CLEM for precise localization of mitochondrial proteins. (A-B) Representative electron micrographs of mitochondria showing MFN-1 immunogold labeling from mouse myotubes. MFN-1 is detected as electron-dense black gold particles (MFN-1–positive puncta). MFN-1 labeling is not uniformly distributed across all mitochondrial profiles but is detectable in the majority of mitochondria, particularly at lower magnifications. Mitochondria undergoing fusion, or having recently undergone fusion, display an increased density of MFN-1–positive puncta. Arrows highlight concentrated gold particle clusters, indicating regions of high MFN-1 protein density. Scale bars A: 0.2 μm; B: 200 nm. (C) Immunogold labelling of TOM20 showing its localization in HeLa cells using Tokuyasu cryo-sectioning. Gold particles (10 nm) specifically mark the mitochondrial outer membrane (arrows). Scale bar: 5μm. (D) Correlative light and electron microscopy (CLEM) analysis of the cellular distribution of CDK1 in HeLa cells. Red fluorescence signals indicate regions of elevated CDK1 concentration at mitochondria (arrows indicate sites of correlation between fluorescent signals and corresponding ultrastructural features). Blue nuclear staining provides cellular context. Scale bar: 5 μm.

The power of high-resolution TEM for revealing structure-function relationships in mitochondria is exemplified by the work of Cogliati and colleagues, who used TEM to demonstrate that OPA1-dependent cristae remodeling influences the assembly and stability of respiratory super complexes, providing a mechanistic link between mitochondrial ultrastructure and bioenergetic efficiency (Cogliati et al., 2013). TEM has also produced insights into how MERCs are organized and physically interact with the endoplasmic reticulum *via* known tethering proteins (Fig. 2A-H). For example, TEM was used in conjunction with the manipulation of the MERC-associated protein mitofusin-2 (MFN2), which is affected in several neurological diseases, to investigate how the structural and functional organization of MERCs is altered under pathophysiological conditions (Han et al., 2021). TEM has also provided detailed ultrastructural views of MDVs, particularly in models of mitochondrial damage (Cadete et al., 2016). Furthermore, immunogold labelling in TEM allows precise localization of specific proteins within mitochondrial sub-compartments (Vogel et al., 2006). Using antibody-conjugated gold particles, proteins such as mitofusin-1 (MFN1) (Fig. 3A–B) and the mitochondrial import receptor subunit TOM20 (Fig. 3C) can be clearly identified on the outer mitochondrial membrane.

Despite these strengths, TEM has several key limitations. Sample preparation can introduce artifacts, particularly in membrane structures (Winey et al., 2014b). Furthermore, TEM images are inherently two-dimensional, making it challenging to interpret complex three-dimensional structures such as mitochondrial networks using this method (Holcomb et al., 2013; Neikirk et al., 2023). To mitigate these issues and ensure reliable quantification, standardized protocols for sample preparation, image acquisition, and analysis are crucial. Stereological methods that provide unbiased 3D estimates from 2D TEM images (Fig. 2), although more time-consuming, offer advantages over simple 2D analysis (Duranova et al., 2020). Additionally, complementary 3D EM techniques (e.g., serial block-face scanning EM, discussed further below) can provide a more comprehensive view of cellular ultrastructure (Fig.2I) (Marshall et al., 2023c).

#### Electron Tomography (ET)

ET extends TEM’s capabilities by reconstructing 3D volumes from a series of tilted 2D projections. In ET, TEM images are collected as the sample is tilted through a range of angles (typically ± 60-70 °); these images are then aligned and computationally reconstructed to create a 3D volume (tomogram) that can achieve resolutions of 4-6 nm. (Beck and Baumeister, 2016). ET has been used to study mitochondrial ultrastructure since the late 1990s and produced significant advancements in understanding mitochondrial structure and function in the 2000s (Frey and Mannella, 2000; Mannella, 2006).

ET has provided crucial insights into mitochondrial architecture. For example, it helped show that cristae are not simple invaginations of the IMM but rather complex, interconnected structures with diverse morphologies (Mannella et al., 1994; Perkins et al., 1997). Additionally, ET enabled visualization of cristae junctions and demonstrated how these structures change under different physiological conditions (Perkins et al., 1997). The improved spatial resolution of ET also allowed for MDV formation to be clearly distinguished from mitochondrial fusion/fission events (Cadete et al., 2016).

More recently, cryoelectron tomography (also called electron cryotomography; cryo-ET) has become a powerful tool for studying mitochondrial ultrastructure in a near-native state. Cryo-ET, which flash-freezes samples to preserve their native structure without chemical fixation or staining, has provided unprecedented views of mitochondrial protein complexes *in situ*, including the organization of ATP synthase dimers along cristae edges (Davies et al., 2012). With a resolution of 1–2 nm, cryo-ET has been instrumental in defining the organization of the cristae membrane and mapping the localization of respiratory chain complexes (Dudkina et al., 2011). Cryo-ET employed in the ciliate *Paramecium tatraurelia*, revealed the assembly of ATP synthase dimers into helical structures around the outer perimeter of twisted tubular cristae, demonstrating that ATP synthase dimer assemblies shape cristae morphology (Mühleip et al., 2016; Blum et al., 2019). Furthermore, cryo-ET combined with focused ion beam milling (discussed further below) has enabled imaging of mitochondria in their cellular context, providing insights into their interactions with other cellular structures (Wagner et al., 2020).

These evolving ET techniques continue to push the boundaries of our understanding of mitochondrial structure and function. However, ET is also not without limitations as its limited tilt range can cause an artifact called the ‘missing wedge’ that leads to the artificial elongation of structures and uneven or anisotropic resolution in the reconstructed volume (Zhai et al., 2020). For mitochondria, this artifact can obscure small features such as crista junctions or membrane contact sites and can artificially elongate tubular structures, potentially leading to misinterpretation of their true morphology. Additionally, the sample thickness in ET is typically restricted to less than 500 nm, which can make studying entire mitochondria or mitochondrial networks *in situ* challenging (Rigort et al., 2012). Nonetheless, ET remains a prominent tool in mitochondrial research, providing crucial 3D insights into mitochondrial ultrastructure and function.

#### Volume EM (vEM) in Mitochondria Research

Three-dimensional (3D) EM techniques, often collectively referred to as vEM, have greatly expanded mitochondrial research by enabling the reconstruction of organellar networks and spatial relationships within cells (Baena et al., 2021; Hinton et al., 2023; Katti et al., 2022a). Two key methods – focused ion beam scanning EM (FIB-SEM) and serial block-face scanning EM (SBF-SEM) – are particularly notable for generating high-resolution volumetric data on mitochondrial ultrastructure (Fig. 2I) (Marshall et al., 2023a; Faitg et al., 2025), providing even deeper insights into mitochondrial dynamics, morphology, and interactions with other cellular structures.

#### FIB-SEM

FIB-SEM combines the high-resolution imaging capabilities of scanning EM with precision milling using a focused ion beam, enabling the sequential removal of thin layers (typically 5-10 nm) from a sample. The exposed surfaces are imaged in detail, and the resulting images can be computationally reconstructed into a 3D volume. With resolutions comparable to TEM (5-10 nm), FIB-SEM enables visualization of larger volumes, typically up to several hundred cubic micrometers (Baena et al., 2021; Knott et al., 2008).

FIB-SEM’s ability to mill samples *in situ* eliminates the need for ultrathin sectioning, reducing artifacts associated with mechanical sectioning. This capability has provided insights into the spatial relationships between mitochondria and other structures within their native cellular context, such as between mitochondria and myofilaments in striated muscle cells (Katti et al., 2022b). FIB-SEM was used to create a 3D reconstruction of neural tissue, revealing the heterogeneity of mitochondrial morphology and distribution in different neuronal compartments (Kasthuri et al., 2015). Additionally, FIB-SEM has been instrumental in mitochondrial disease research. In an analysis of skeletal muscle fibers from individuals with mitochondrial DNA mutations associated with mitochondrial myopathy, 3D FIB-SEM reconstructions revealed extensive mitochondrial network remodeling, including the formation of elongated mitochondrial nanotunnels, suggesting potential compensatory mechanisms in response to stress caused by mitochondrial diseases (Vincent et al., 2016).

FIB-SEM has limitations: f or example, the process of sequentially removing sample material is destructive, meaning that once imaged, samples cannot be revisited. The technique is also time-consuming and computationally intensive, especially for imaging large volumes. Moreover, ion beam damage can introduce artifacts, particularly in beam-sensitive biological specimens, such as resin-embedded or cryo-preserved samples, lipid-rich membranes, and protein-dense organelles, where ion milling can cause local heating, mass loss, amorphization, and membrane thinning (Hoffman et al., 2020). Advancements in cryo-FIB-SEM, which enable imaging of samples in a near-native, hydrated state, aim to mitigate some of these issues (Schertel et al., 2013).

#### SBF-SEM

SBF-SEM is another EM technique that generates high-resolution 3D reconstructions of cellular structures, similar to FIB-SEM, but with a different sample sectioning approach (Crabtree et al., 2024; Marshall et al., 2023c). In SBF-SEM, a resin-embedded sample is imaged in an SEM chamber, and after each imaging cycle, an ultramicrotome slices thin layers (typically 25-100 nm thick) from the sample surface (Marshall et al., 2023c). These successive layers are imaged and then reconstructed into a 3D volume. SBF-SEM achieves lateral resolutions of 10-20 nm and can image volumes up to several hundred cubic micrometers.

SBF-SEM is particularly useful for studying mitochondrial morphology and distribution across different cell types and tissues (Fig. 2 I, J). For example, SBF-SEM was used to investigate changes in mitochondrial morphology and function associated with aging or loss of the MICOS complex in muscles and cardiac tissues (Vue et al., 2023). SBF-SEM has been used to generate detailed 3D reconstructions of mitochondrial networks in muscle, revealing intricate connections between mitochondria and the sarcoplasmic reticulum in human skeletal muscle fibers (Hinton et al., 2024) and highlighting an intricate mitochondrial network with extensive inter-mitochondrial junctions in cardiac muscle (Pinali et al., 2013). These findings suggest that the spatial organization of mitochondria underpins functional coupling in muscle energy metabolism by supporting efficient energy transfer within muscle cells, allowing ATP production to match local metabolic demand.

Combining SBF-SEM with genetic and biochemical analyses has also proven beneficial in studying mitochondrial behavior in disease states. For instance, as discussed above, Vincent and colleagues employed both FIB-SEM and SBF-SEM to reveal previously unrecognized patterns of mitochondrial network remodeling in individuals with mitochondrial DNA disorders (Vincent et al., 2016), demonstrating the potential of these techniques to advance our understanding of mitochondrial diseases.

Despite its advantages, SBF-SEM also has limitations. Compared to FIB-SEM, its z-axis resolution is lower because comparatively thicker sections are removed during imaging. Additionally, like FIB-SEM, it is a destructive technique, and the time required for large-volume acquisitions can be substantial. Furthermore, aspects of sample preparation, particularly heavy metal staining and resin embedding, can introduce artifacts that must be considered during data interpretation (Titze and Genoud, 2016).

#### Correlative light and EM (CLEM) in Mitochondrial Research

CLEM combines the strengths of fluorescence microscopy with the ultrastructural resolution of EM, enabling the correlation of fluorescently labeled cellular processes captured either in live cells or after fixation with high-resolution EM imaging of the same structures (Marshall et al., 2023c). An important advantage of CLEM is its compatibility with advanced imaging modalities, including FIB-SEM, TEM, total internal reflection fluorescence (TIRF) microscopy (see Box 1), cryo-EM, SBF-SEM, STED, and SIM (Marshall et al., 2023b). Additionally, CLEM can be combined with software that localizes and correlates ultrastructural components with specific proteins or structures (Fig. 3D), providing detailed insights into their interactions (Kobayashi et al., 2016; Schellenberger et al., 2014).

A major challenge in CLEM is precisely correlating light and EM data. This challenge was addressed by using fluorescent fiducial markers visible under both light microscopy and EM, which provide a reliable coordinate system that allows accurate mapping of positions from fluorescence microscopy images to EM images with a correlation precision of tens of nanometers (Kukulski et al., 2011). This approach has opened new possibilities for studying ultrastructures associated with dynamic cellular processes. However, fiducial markers can interfere with cellular processes, necessitating careful control experiments. The remaining challenge for the application of CLEM in mitochondrial research lies in maintaining sample integrity during the transition from live-cell imaging to EM preparation, underscoring the need for rapid, gentle fixation protocols (Karreman et al., 2016; Kolotuev et al., 2009). As in TEM, chemical fixation methods can introduce artifacts (Winey et al., 2014a); however, improvements in freeze-substitution and embedding protocols that work well for both fluorescence microscopy and TEM, in turn benefiting correlative imaging techniques, have been developed (Karreman et al., 2012). This method significantly improved preservation of mitochondrial ultrastructure while maintaining fluorescence signals, although some unavoidable structural alterations, such as subtle membrane shrinkage, cristae distortion, and partial lipid extraction, remained. Such effects can alter the apparent shape or spacing of mitochondrial membranes, complicating quantitative ultrastructural analysis.

Despite these challenges, CLEM has emerged as a powerful tool, particularly for studying dynamic processes such as mitochondrial fission, fusion, and mitophagy (Cornelissen et al., 2018; Godtliebsen et al., 2023; Jung et al., 2020; Li et al., 2021; Munson et al., 2021; Zhen et al., 2020). For example, CLEM was recently employed to investigate ultrastructural changes associated with mitochondrial fission, which were shown to be coordinated by the combined roles of Mitofusin/Atg44 and dynamin-related protein/Dnm1 (Furukawa et al., 2024). Similarly, CLEM was used in SH-SY5Y neuroblastoma cells to track mitophagy induced by the mitophagy modulator propionic acid to study its potential mechanisms in neurodegenerative diseases (Jung et al., 2020). However, it should be noted that the delay between live-cell imaging and sample fixation (approximately 5-10 seconds) can still miss rapid structural changes (Fermie et al., 2018; Kukulski et al., 2011) such as those that might occur during fission, fusion, and mitophagy.

#### Cryo-CLEM

Compared with CLEM, cryo-CLEM minimizes chemical fixation and dehydration artifacts, providing a more accurate representation of cellular structures. Schellenberger and colleagues pioneered cryo-CLEM by developing a workflow to study the trafficking of fluorescent particles within cells (Schellenberger et al., 2014). This technique was applied to correlate super-resolution fluorescence microscopy of mtDNA with cryo-ET, revealing the native organization of nucleoids within mitochondrial ultrastructure and demonstrating the potential of cryo-CLEM to provide molecular-resolution insights into mitochondrial biology (Kukat et al., 2011; Jakobs & Wurm, 2014; Stephan et al., 2019). However, technical challenges remain, such as maintaining vitreous ice during fluorescence imaging and the limited penetration depth of cryo-ET. (Last et al., 2023; Lucić et al., 2005)

A cryo-CLEM approach compatible with focused ion beam milling that allows targeted imaging of specific cellular regions identified by fluorescence microscopy was recently developed, thereby making cryo-CLEM applicable to thicker cellular specimens (DAM et al., 2021; Hsieh et al., 2014). Using this approach, a study of MERCs in yeast (referred to as ER-mitochondria encounter structures, ERMES) resolved the internal supramolecular architecture of the lipid transfer machinery at ERMES, unveiling lipid flux mechanisms in eukaryotic cells (Wozny et al., 2023). The further integration of CLEM with other advanced imaging techniques holds promise for future mitochondrial research. For instance, cryo-CLEM was combined with cryo-focused ion beam milling and cryo-ET to study mitochondrial cristae remodeling during apoptosis (Ader et al., 2019). Despite these advancements, the complexity of this technique limits its throughput and constrains its application to specialized research facilities, underscoring the need for further methodological refinement and technological developments to improve accessibility.

#### Volume Correlated Light and EM (vCLEM) in mitochondrial research

Volume-CLEM or vCLEM integrates the strengths of CLEM with vEM techniques to investigate cellular physiology at high-resolution ultrastructural detail throughout entire cell volumes (Peddie et al., 2017). This approach enables 3D visualization of protein–ultrastructure relationships and cellular connectomics, allowing researchers to identify complex architectures and interactions within the densely packed intracellular environment (Peddie et al. 2017; Hoffman et al. 2020). This makes vCLEM especially valuable for assessing rare and transient contacts between the mitochondrial membrane and the ER (Jung and Mun, 2019), lysosomes, and possibly other organelles. Additionally, vCLEM has been used to visualize transient changes in mitochondrial morphology due to loss of mitochondrial membrane potential and serves as an important tool for directly correlating these changes with cellular dysfunction (Miyazono et al., 2018). The minutiae of methodology for vCLEM vary depending on imaging goals, but it is applicable across different formats and customizable. Current literature suggests that the use of vCLEM would be ideal for visualizing fine details of transient but integral cell-cell connections and for understanding the correlative value and consequences of changes to a capricious mitochondrial environment.

#### Expansion Microscopy i n Mitochondrial Research

**Fig. 4:**
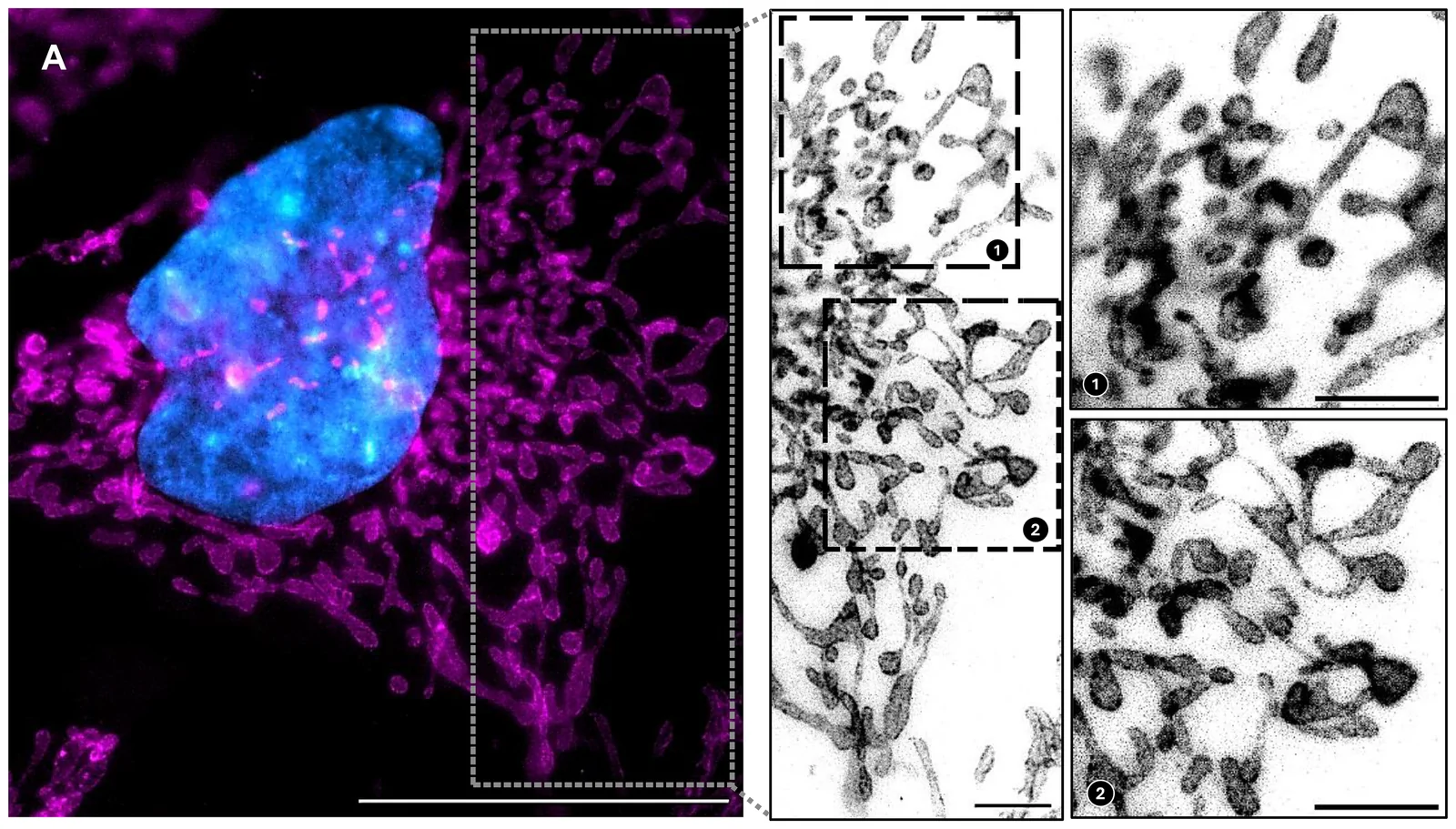
Nanoscale organization of TOM20 clusters revealed by TR-Expansion Microscopy. Maximum-intensity projection of TOM20 (red) across the mitochondrial network visualized by expansion microscopy. Physical expansion of the specimen (8.5×) enhances spatial resolution, revealing the detailed organization and clustering of TOM20 along individual mitochondria. The nucleus is labeled with Hoechst (cyan). Dashed boxes indicate regions shown as higher-magnification inset images on the right. Images were acquired using a THUNDER imager with a 40× objective. Scale bar, 5 µm (colored overview image); 1 µm (inset images), adjusted for the 8.5× expansion factor.

Expansion microscopy (ExM) has rapidly become a transformative technique for investigating mitochondrial ultrastructure and protein organization at nanoscale resolution. By physically expanding biological specimens while preserving their relative spatial organization, ExM enables imaging nanoscale structures with conventional microscopes, overcoming the diffraction limit of light without the need for advanced super-resolution systems (Chen et al., 2015; Humpfer et al., 2024; Wassie et al., 2019). ExM is thus particularly relevant for mitochondrial research, where understanding the intricate details of cristae and protein distributions is crucial for comprehending mitochondrial function and dysfunction (Fig. 4).

ExM works by embedding samples in a swellable polymer, digesting cellular components to facilitate expansion, and then imaging the expanded samples at higher resolution using standard microscopes. Expansion factors typically range from 4x to 10x (Chen et al., 2015; Chozinski et al., 2016; Humpfer et al., 2024; Wassie et al., 2019). Methodological variants, such as protein retention ExM (proExM), in which proteins are anchored to the swellable gel, allow for higher-fidelity imaging of mitochondrial structures (Tillberg et al., 2016). Furthermore, ultrastructure ExM (U-ExM), developed by Gambarotto and colleagues, further refined this approach, achieving improved protein retention and uniform isotropic expansion, making it an ideal tool for mitochondrial studies (Gambarotto et al., 2019a). A key consideration when evaluating ExM approaches is the choice of anchoring chemistry. For example, proExM primarily relies on pre-expansion labelling, in which proteins are anchored to the polymer using Acryloyl-X (AcX), although post-expansion antibody labeling is possible under modified homogenization conditions. In contrast, techniques such as fluorescent labeling assisted by stimulated hydrolysis (FLASH) enable systematic post-expansion labeling of mitochondrial proteins, broadening the scope of ExM applications. (Tillberg et al., 2016; Di Gallo et al., 2025)

ExM and its variations have provided exciting insights into mitochondrial ultrastructure. Combining ExM with STED microscopy (ExSTED) achieved an effective resolution of ∼15 nm, revealing fine-scale variations in cristae morphology that correspond to distinct mitochondrial metabolic states (Gao M et al., 2021). Similarly, ExM was used to visualize the mitochondrial internal structure and characterize mitochondrial cristae morphology by labelling the intermembrane space (Kunz et al., 2020). The same approach also enables nanoscale mapping of multiple mitochondrial proteins relative to cristae organization in fixed cells (Kunz et al., 2020). Furthermore, a novel ExM protocol, termed Ten-fold Robust Expansion Microscopy (TREx), which enables a ten-fold expansion of both thick tissues and cultured cells, allows ultrastructural detailing of subcellular protein localization by combining antibody labelling with small-molecule stains to label total protein and membranes (Damstra et al., 2023). The integration of ExM with other advanced microscopy techniques, such as single-molecule localization microscopy (SMLM) (Zwettler et al., 2020), continues to push the boundaries of mitochondrial research, making ExM a powerful tool for both structural and functional investigations. Recently, an iterative ultrastructure expansion microscopy (U-ExM) protocol that enables expansion up to 26X was developed. U-ExM coupled with cryofixation was able to resolve individual cristae and observe a crista spacing of about 85 nm (Gambarotto et al., 2019; Louvel et al., 202 3).

## Conclusions

Microscopy continues to drive advances in mitochondrial research, revealing intricate structural details and critical functional roles of these organelles within cellular systems. Technological innovations, from initial EM observations to contemporary breakthroughs in super-resolution imaging, ET, and ExM, have fundamentally transformed our understanding of mitochondrial dynamics, mitochondria-organelle interactions, and bioenergetic processes. Today, integrating advanced techniques, including cryo-FIB-SEM and *in situ* cryo-ET, enables structural analysis of mitochondria within near-native cellular environments, and live-cell super-resolution microscopy facilitates real-time visualization of mitochondrial processes at molecular resolution, effectively bridging structure-function relationships. Looking ahead, machine learning approaches are expected to accelerate discovery by supporting automated analysis of complex imaging datasets. Such methods may enable robust segmentation of mitochondrial networks in challenging imaging conditions and provide new avenues for classifying dynamic fusion and fission events alongside morphology changes linked to mitochondrial function.

Despite these remarkable achievements, advanced mitochondrial imaging still faces critical constraints, including the complexity of multidimensional data arising from fusion-fission dynamics, phototoxicity-induced effects on membrane potential that impact measurements using fluorescent dyes, and systematic imaging artifacts that affect network topology analysis. Infrastructure requirements, the need for access to specialized facilities, and substantial costs limit the widespread adoption of these techniques across research institutions. Addressing these methodological limitations requires systematic interdisciplinary collaboration: physicists to optimize adaptive optics for mitochondrial morphologies, computer scientists to develop automated segmentation algorithms for organelle networks, and chemists to engineer photostable mitochondria-targeted probes with reduced cellular toxicity. By integrating strategic expertise, researchers can achieve greater resolution in the study of mitochondrial structure-function relationships, facilitating transformative discoveries in cellular bioenergetics and mechanisms of metabolic disorders that will advance our fundamental understanding of organelle biology and disease pathogenesis.

## Summary

In this Review, the authors provide an overview of how advanced microscopy techniques reveal the intricate ultra structure, dynamics, and interactions of mitochondria, enhancing our understanding of their essential roles in cellular metabolism, aging, and disease.

## Box 1 Fluorescence microscopy methods for assessing mitochondrial structure-function: advantages and disadvantages

### Conventional Fluorescence Techniques

**Widefield Fluorescence Microscopy** (∼200 nm lateral/∼600 nm axial) utilizes uniform specimen illumination with broad emission collection and enables rapid acquisition with minimal photodamage, facilitating long-term monitoring of mitochondrial morphology, membrane potential, and distribution, although it is limited by out-of-focus signal in thick samples (Jakobs et al., 2020; Loew et al., 1993)

**Confocal Laser Scanning Microscopy (CLSM)** (∼180 nm lateral/∼500 nm axial) employs point-scanning with pinhole apertures to reject out-of-focus light, providing superior optical sectioning for reconstruction of three-dimensional mitochondrial networks (Collins, 2002; Katti et al., 2022a). CLSM offers enhanced contrast but introduces increased photodamage and reduced temporal resolution.

**Spinning Disk Confocal Microscopy** (∼200 nm lateral/∼500 nm axial) uses parallel scanning through multiple pinholes on a rapidly rotating disk, enabling simultaneous multi-point illumination and detection (Nakano et al., 2002). A nipkow spinning-disk confocal system was employed to visualize mitochondrial dynamics in real time, which enabled the high-speed imaging of fluorescent proteins in individual mitochondria (Liu et al., 2009).

**Multiphoton Microscopy** (∼300 nm lateral/∼800 nm axial) excites via simultaneous absorption of multiple lower-energy photons, confining excitation to the focal plane. It provides superior tissue penetration (up to 1 mm) with reduced phototoxicity, enabling visualization of mitochondria in intact tissues and assessment of metabolic activity through endogenous fluorophores. (Helmchen and Denk, 2005; Zipfel et al., 2003).

### Super-Resolution Techniques

**Stimulated Emission Depletion (STED) Microscopy** (∼30-80 nm lateral/∼500 nm axial) employs a donut-shaped depletion beam to selectively deactivate fluorophores at the periphery of the excitation focus, enabling direct optical super-resolution visualization of nanoscale protein distributions, cristae structure, and nucleoid organization, although it requires specialized fluorophores and higher illumination intensities. (Jakobs et al., 2020; Stephan et al., 2019a)

**Single-Molecule Localization Microscopy (PALM/STORM)** (∼10-30 nm lateral/∼50 nm axial) achieves super-resolution by sequentially activating, localizing, and bleaching sparse subsets of fluorophores. These methods provide the highest resolution among fluorescence microscopy techniques with quantitative capabilities for precise protein mapping and nucleoid structure analysis, but require specialized probes and extended acquisition times. (Appelhans et al., 2012; Betzig et al., 2006; Huang et al., 2008; Rust et al., 2006)

**Structured Illumination Microscopy (SIM)** (∼100 nm lateral, ∼300 nm axial) uses patterned illumination and computational reconstruction to extract high-frequency spatial information. SIM offers an optimal balance between spatial and temporal resolution for visualizing cristae dynamics and fusion-fission events in living mitochondria. (Huang et al.,2018)

**Image Scanning Microscopy (Airyscan)** (∼140 nm lateral/∼400 nm axial) captures the complete Airy disk pattern using a detector array with computational reassignment of pixels, providing moderate resolution enhancement with significantly improved signal-to-noise ratio and reduced illumination requirements for extended imaging of mitochondria (Kolossov et al., 2018).

### Functional Techniques

**Total Internal Reflection Fluorescence (TIRF) Microscopy** (∼200 nm lateral/∼50-100 nm axial) selectively excites a thin (∼100 nm) region adjacent to the coverslip using an evanescent wave. TIRF offers a superior signal-to-noise ratio for visualization of mitochondria-membrane interactions and early mitophagy events, although its range limits imaging to mitochondria at the cell periphery (Axelrod, 2001; Kudalkar., et al 2016)

**Light Sheet Fluorescence Microscopy** (∼300-500 nm lateral/∼1 μm axial) uses orthogonal illumination with a thin sheet of light and wide-field detection, dramatically reducing phototoxicity while enabling rapid volumetric acquisition, enabling extended imaging of mitochondrial dynamics in developing embryos and large specimens. (Power and Huisken, 2017)

**Förster Resonance Energy Transfer (FRET) Microscopy** (∼200-250 nm lateral/∼500-700 nm axial) detects energy transfer between donor and acceptor fluorophores in close proximity (1-10 nm). FRET can reveal molecular interactions and conformational changes within mitochondrial proteins, providing functional information beyond structural data. (Sun et al., 2013; Wallrabe and Periasamy, 2005)

**Fluorescence Lifetime Imaging Microscopy (FLIM)** (∼200-250 nm lateral/∼500-700 nm axial) measures the lifetime of excited fluorophore states rather than intensity, allowing concentration-independent assessment of mitochondrial metabolic state, pH, and membrane properties through environment-sensitive lifetime measurements. (Skala et al., 2007; Van Munster & Gadella, 2005)

**Photobleaching Techniques (Fluorescence Recovery After Photobleaching, FRAP; Fluorescence Loss In Photobleaching, FLIP)** (∼200-250 nm lateral) analyze fluorescence recovery (FRAP) or loss (FLIP) patterns following controlled photobleaching. These methods enable quantitative assessment of protein diffusion rates, membrane fluidity and matrix connectivity within mitochondria (Lippincott-Schwartz et al., 2001; Reits and Neefjes, 2001; Simula & Campello, 2018; Jakobs S. 2006 and Partikian et al., 1998).
